# Speed of Processing and Personality: The Influence of Personality and Extrinsic Feedback on the Performance of Cognitive Tasks

**DOI:** 10.3390/bs10040076

**Published:** 2020-04-08

**Authors:** Ming Yu Claudia Wong, Pak Kwong Chung, Ka Man Leung

**Affiliations:** 1 Department of Sport and Physical Education, Hong Kong Baptist University, Kowloon, Hong Kong, China; pkchung@hkbu.edu.hk; 2 Department of Health and Physical Education, The Education University of Hong Kong, Hong Kong, China; leungkaman@eduhk.hk

**Keywords:** personality, feedback, motivation, conscientiousness, extraversion

## Abstract

Background: Feedback is considered as an effective means of motivating, guiding, and reinforcing desired behaviours. However, the ways to interpret external feedback may be different among individuals with different personality traits; therefore, this would influence the effects of feedback on performance. Accordingly, the influencing effects of personality towards different styles of feedback on cognitive task performance were examined. Methods: Participants (N = 71) were given three Stroop tasks as a dependent variable, whereas the Trail Making Task was an independent variable; additionally, a personality test was used to record the personality traits of each participant. The relationship between personality and feedback-induced changes in Stroop performance was computed by means of Pearson correlation, followed by a mixed-effect model to demonstrate the effect of personality on the overall performance with feedback. Results: The statistical analysis indicated that performance from those with higher levels of extraversion generally profitted from feedback, irrespective of whether it was negative feedback (r = 0.201) or positive feedback (r = 0.205). Additionally, the moderating effect of personality on feedback and performance was demonstrated. Conclusions: The limitations of the sample size and other external influences may have reduced the representativeness of the research. Nonetheless, more potential influencing factors need to be included and explored in future research.

## 1. Introduction

Feedback is considered to be an important psychological concept that influences an individual’s development. It refers to a process of receiving and inputting from the external environment based on the actions or output, either in a positive or negative way [1]. Given that feedback is provided to guide, motivate, and reinforce task-relevant behaviours, according to the feedback intervention theory [2], the comparison of feedback, desired goals, or standards regulates individual’s behaviour in a hierarchical format. However, the functions and results of feedback manifest differently in different circumstances. The control theory [3] posits that positive feedback can increase deviation and provide an encouraging movement from the individual’s initial self, whereas negative feedback may reduce error or deviation compared to an individual desired goal [4]. However, interpretations of feedback are distinctive; hence, the style of feedback may have different impacts upon different individuals. Positive feedback allows a person to pursue a high standard and increase their effort, or possibly reduce their effort, whereas people who receive negative feedback may further increase their effort to avoid a reduction of achievement and a discrepancy between the desired and actual performance. However, if both styles of feedback bring about no differences in performance or individuals do not pursue a change in discrepancy, then that result is related to individual differences. Some low achievers have been found to gain an enhancement in their self-focus attention, even if the feedback received has contradicted their performance history [5]; in other words, individuals strive for their own goals and redirect their attention in spite of being given negative feedback. Particular individuals tend to attempt improvements in performance and reduce the feedback-targeted discrepancy. Thus, showing the interaction between feedback motivation and individual differences is considered to be valid. 

According to behaviourism, a positive feedback intervention is a function used for reinforcement, and a negative feedback intervention is used for punishment—both facilitate learning and enhance performance. However, previous empirical research yielded inconsistent results regarding the learning theory of behaviourism, suggesting that there is a subtler process in action. Through the use of a feedback loop within learning and motivational models, DeNisi and Kluger (2000) first showed that people learn from their outcomes and that their performance depends on their performance feedback [6]. The authors assumed that job or task performance will improve as a result of feedback, especially when compared with the performance of those who did not receive any feedback. However, a meta-analysis showed that only less than one-half of the overall sample experienced positive effects under the intervention of feedback [6]. This led to an argument regarding whether receiving negative or positive feedback is a critical factor in determining a person’s reaction. Thus, this raised Hypothesis 1a—prior positive feedback will facilitate performance on a subsequent task—and Hypothesis 1b—prior negative feedback will impair performance on a subsequent task. Moreover, considering that experimental design research may consist of various possible confounding variables or limitations, more “evidence-based” experimental research is needed on the effects of negative feedback, including research testing other possible impact factors, such as individual differences, i.e., personality.

The Big Five Personality Traits model [7]—openness, conscientiousness, extraversion, agreeableness, and neuroticism—has been found to be a good predictor of patterns of behaviour [8], while personality can be used to understand the influences of a person’s thoughts and behaviours [7]. In the conception of personality traits, researchers have suggested that traits are stable over time and consistent across cultures. Human personalities consist of a core consistency and are defined as the “true nature” of individuals [9], despite individuals’ behaviours varying under different occasions and situations. However, the interaction effect of personality and situation behaviours are supported by the cognitive–affective personality system [10] and the personality–situation interactional theoretical model [11], which illustrated that the relationships between the big five traits, behaviours, and task performance are affected by the situation or task context, as well as the outcomes of the task, such as the impact of the acts and the obtaining of errors and their consequences. Furthermore, personality-to-achievement associations may emerge as a result of direct effects of personality on performance [12]; these indicate that different task performance variables, such as task effectiveness and achievement outcomes, are involved in different personality traits, and this occurs through the processing of information. In other words, it can be assumed that a person’s interpretation of their received feedback and achievement may differ due to their contrasting personality. Several research works have illustrated the process of interpreting feedback for particular personality traits; for instance, conscientious people exhibited a high level of self-deception, which largely decreased the feedback effect, and highly conscientious people were therefore more vulnerable to experiencing feelings of tension or frustration to achieve the goal when facing difficulties [13]. In contrast, people with a higher level of neuroticism tended to achieve lower accuracy in repeated tasks after error feedback was given [14]. This raised Hypothesis 2—higher levels of conscientiousness will be associated with positive responses to negative feedback—and Hypothesis 3—higher levels of neuroticism will be associated with poorer performance following negative feedback. Moreover, extraverts showed better emotional management [15,16], and thus could maintain a positive mood after negative stimuli and show a lower decrement in task performance [17]. This raised Hypothesis 4—a higher level of extraversion will result in a lower decrement in performance than lower-level extraverts after receiving negative feedback.

Based on the respective associations between feedback and performance, as well as the relationship of personality with information interpretations and situations, it can be assumed that personality potentially affects the association between feedback and performance. Thus, this research aimed to investigate whether the effect of different styles of feedback—giving positive or negative feedback—on performance will be moderated by an individual’s personality. It is also important to illustrate which style of feedback or motivation method is more effective in improving people’s performance, in addition to parsing out possible influential factors, such as demographic variables and experimenter authority, which may affect a person’s performance differently for individuals with different personality traits. 

## 2. Materials and Methods

### 2.1. Study Design

A correlational repeated measures study design was employed within this study, in which participants experienced both positive and negative feedback during the experiment. This research required participants to be age sixteen or above, with no intellectual disability and no cognitive or colour vision-related illness. This study was located in Edinburgh, Scotland, and the recruited participants consisted of a variety of ethnicities. Level 2 ethical approval was granted by the Department of Clinical and Health Psychology ethics research panel of the School of Health and Social Sciences of The University of Edinburgh (CLIN527). A risk assessment form was also included, ensuring the safety of the researchers and participants within the experiment setting. Under the research ethics and conduct requirements, it was necessary for the participants to complete and sign a consent form with the participant information sheet before the experiment. As the experiment was conducted using the Open Sesame software, researchers needed to make sure that participants wore their glasses or contact lens if necessary. Also, the researchers needed to measure the eye distance between the participants and the computer. All participants were required to complete the experiment within the standardized distance (30 cm). The detailed procedure of the psychological experiment will be shown in Table A1 in Appendix A.

### 2.2. Participants

The sample size was calculated by using the G*Power calculation method [18], with the standard α = 0.05 and power (1−β) = 0.80 [19] and by combining the average effect size of two related studies on personality effect and feedback performances [20,21] at 0.326. The G* Power calculation (correlation test) suggested a sample size of 68 people, with the degree of freedom at 66. In the current study, seventy-one participants took part in the study. One participant was excluded as the score on the Stanford Sleepiness Scale (SSS) was above the cut-off of five. This left a sample with a gender distribution of 46 females, 23 males, and 1 other. Mean age was 31.2 years (range 21–74 years). Employment status was: paid (14 participants), voluntary (1), student (48), retired (7). 

### 2.3. Outcome Measures

The outcome measures used in the present study were the Stroop tasks, with the given feedback as the independent variable and the personality test as the predictor.

Stroop task [22]. The Stroop effect is one of the longest-standing and best-known phenomena in cognitive science and psychology [23]. The basic form of Stroop is to name the colour of the ink in which a word is printed, whilst ignoring the actual word itself. Given the Stroop task being a dependent variable in this study, it acted as a tool to measure a person’s of interference control through examining the speed of information processing. It enabled the current experiment to identify the change of information processing speed after a person receives different feedback. 

Trail Making Test [24]. The Trail Making Test is being suggested as being effective in differentiating between individuals and patients with and without brain injury [25] by measuring cognitive processing speed in visual speed tasks [26,27]. The trail making test was set as a tool to provide feedback to the participants, while the results of the test were not included in the research. 

Feedback. Feedback, acting as an independent variable, was given after the completion of a trail making test. The general positive and negative feedback was given to the participants through a visually shown normal distribution, which stated the average performance level of 64 healthy adults and the researcher stated that “According to the normal distribution statistic, on this task, you performed better (or worse) than 75% of all the participants in this study so far”, regardless of the participants’ performance. Despite the fact that the feedback given was not true from the exact performance, a debriefing was given after the end of the experiment. 

The International Personality Item Pool Presentation of the NEO PI-R (IPIP-NEO 120) [28]. A personality test was given to indicate another independent variable—personality traits. In this research, the shorter version with 120 items of IPIP-NEO was adopted in the questionnaire. Maples et al. (2014) have examined the reliability, and convergent and criterion validity of the 300-item IPIP-NEO, comparing it with the 120-item version. Both scales demonstrated strong reliability and convergence with the NEO PI-R. They also showed a significant similarity of correlational profiles across the criterion variables (rICC = 0.983, 0.972, and 0.976, respectively) [29]. Therefore, the statistical results proved that the 120-item version of IPIP-NEO could be used as a valid assessment tool for measuring the big five personality traits.

Stanford Sleepiness Scale (SSS) [29]. The Stanford Sleepiness Scale is a self-report scale that asks the respondent to evaluate their current level of sleepiness. Through the scale, people’s sleepiness can be quantified and show correlation with different psychological perspectives. Hoddes et al. (1973) quantified the relationship between sleepiness and performance on mental tasks. In their research, it showed that the mean SSS ratings correlated with performance on the cognitive test— the Wilkinson Test—with r = 0.68. Also, it correlated with performance on the cognitive memory test, with r = 0.47. According to Balkin et al. (2004), the effects of sleep loss will make people less sensitive when operating tasks and resulting in performance degradation. Their research results proved that those in the group with restricted sleep performed poorly compared to those with standardized 8 hours of sleep. In addition, their performance also improved after recovery. Within this study, responses of 5 or above on the SSS (which corresponded to the statement: “Foggy; losing interest in remaining awake”) resulted in their data being excluded from the study.

### 2.4. Statistical Method

A descriptive analysis of participants’ demographic information, the overall average feedback performance, and the personality statistics were done. A correlation analysis has been done to indicate the relationship between personality, demographic variables, experimenter effect, and feedback. Moreover, a mixed-effect model has been shown to interpret the effect of personality on the overall performance after feedback, at the same time, acting as a tool to verify the validity of the sample data. 

## 3. Results

### 3.1. General Findings 

The mean of three different response times—after neutral (without feedback), positive feedback, and negative feedback Stroop tasks—are shown. Regardless of different personality traits, the average response time of the Stroop task after negative feedback (M = 876.06, sd = 2407.02) was higher than that of positive feedback (M = 866.35, sd = 1458.89). This illustrated that most people reacted slower after receiving negative feedback.

### 3.2. Major Findings

#### 3.2.1. Hypothesis 1a

**Hypothesis** **1a.** *Prior positive feedback will facilitate performance on a subsequent task.*

**Hypothesis** **1b.** *Prior negative feedback will impair performance on a subsequent task.*

Responding to H1, a paired t-test (Table 1) showed no significant differences between the performance after positive and negative feedback. Despite the fact that the statistics showed significant differences between performance with and without feedback, and the response time results after positive feedback were slightly faster than that of negative feedback, it could not identify that negative feedback would necessarily impair the performance, thus the null hypothesis could not be rejected. 

#### 3.2.2. Hypothesis 2

**Hypothesis** **2.** *Higher levels of conscientiousness will be associated with positive responses to negative feedback.*

**Hypothesis** **3.** *Higher levels of neuroticism will be associated with the poorer performance following negative feedback.*

A correlation (Table 2) was done to identify the potential factors towards feedback and to justify the significance of the proposed effect direction. As in Table 2, with a 95% confidence interval, both neuroticism (r = −0.11, *p* = 0.183 and r = −0.162, *p* = 0.09) and conscientiousness showed no significant correlation with the mean median response time differences of both positive and negative feedback. Therefore, H2 and H3 were rejected, given that the performance of conscientious people did not show any association with the received feedback and neuroticism was shown to be less likely to be influenced or overwhelmed by the effect of feedback. 

#### 3.2.3. Hypothesis 4

**Hypothesis** **4.** *A higher level of extraversion will show less decrement in performance than those of lower extraverts after receiving negative feedback.*

**Figure 1:**
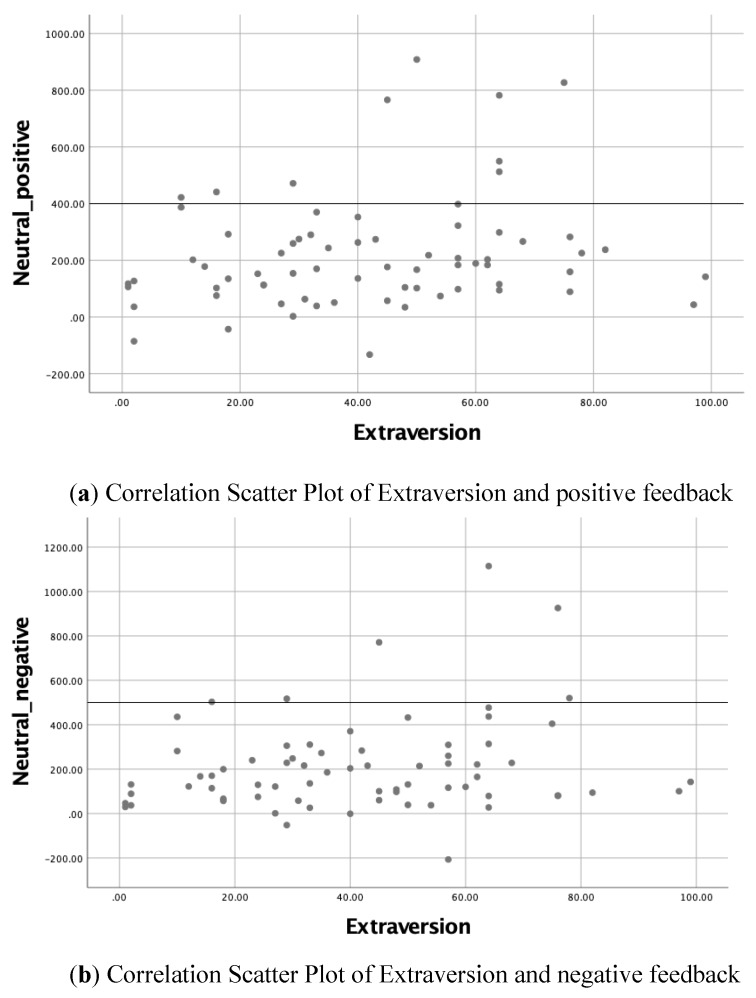
Correlation scatter plot of extraversion and style of feedback. (**a**) Correlation Scatter Plot of Extraversion and positive feedback, (**b**) Correlation Scatter Plot of Extraversion and negative feedback.

Lastly, given that the extraverts’ neutral-negative median differences were slightly lower than that of other personality traits, this represents that extraverts were less impaired by negative feedback. Extraverts also showed a significant positive correlation (Table 2) with both neutral-positive and neutral-negative median response time differences (r = 0.205, *p* = 0.044 and r = 0.201, *p* = 0.047, respectively). This could only indicate that a higher level of extraverts would profit from feedback irrespective of the style of feedback, thus they were less overwhelmed by feedback. Hence, the null hypothesis of H4 could not be rejected. Despite this, the correlation scatter plot shown in Figure 1 illustrates that the correlation effect was mainly influenced by a few participants, which the result may consider as less representative, and there were participants’ performances along the slopes. 

### 3.3. Other Influences and Validity of the Sample

The correlation matrix (Table 2) showed that the demographic variables were not significantly correlated with feedback performance. Therefore, they were not considered as influential under this research. Moreover, agreeableness showed a significant negative correlation with positive feedback performance, with r = −0.212, *p* = 0.039. Furthermore, this research has shown a relatively low trade-off between accuracy and speed. The accuracy record showed that nearly 85% of the participants got 100% correct in both task, irrespective of the style of feedback; while the rest of the 10% scored 14 to 15 correct out of 16. 

On the other hand, despite the fact that the literature has reviewed the personality-to-achievement association, the regression analysis on the direct effect of personality on the raw test response time showed no significant results (Table A3). Upon that, a mixed-effect model was done to demonstrate the effect of personality on the overall performance with feedback and to verify the representativeness of this sample on presenting the relationships by controlling the interactive effect between personalities. The personality trait variable showed a significant effect on the overall performance with feedback, with F(2,140) = 3.609, *p* = 0.009. Despite that, with the significant correlation and mixed-effect model, we can still indicate the possible relations between the research variables. 

## 4. Discussion

The research was launched to investigate the influencing effect of personality on the performance outcome with the intervention of different kinds of feedback. The current research could not affirm that negative feedback will impair performance on a subsequent task, and high extraversion was shown as benefiting from feedback, irrespective of the style. However, the results have shown no support on the effect of extraversion and neuroticism on feedback performance in the present study. Moreover, agreeableness was shown as benefiting from the positive feedback, given that people with a high level of agreeableness had performed better with positive feedback. At last, the mixed-effect model demonstrated that personality traits showed a significant effect on overall performance changes after feedback was given. 

Personality and Performance. The way feedback is presented towards individuals with different personality types may cause diverse results. Matthews G. and his team (2003) have suggested that personality is one of the factors that moderates the feedback effect of performance—personalized feedback to individuals can raise the function of feedback to a larger extent. In his research, he has indicated that the neutral feedback slant—statements with no subjective judgement towards participant’s performance and with no bias [9]—in particular, has shown significant effect towards all high conscientious participants, including those who scored a lower mark. Studies have also shown that people with a high level of agreeableness tend to achieve better performance outcomes, as well as having a strong affective reaction on feedback and feedback acceptance [30,31,32], thus they would perform better under the feedback condition. The indication of neuroticism (H3) was regarded as considerable, and extraverts were shown to benefit in performance after receiving feedback, either positive or negative. It documented the consequences of personality towards the effect of feedback on performance outcome. Extraverts indicated significant positive affect reaction and confidence on performance and achievement [33]. Whereas, Waterston (2014) opposed the description of extraverts as optimistic and outgoing. In fact, they are quite emotional and the results in emotional intelligence research are varied [34], while people with neuroticism have shown lower emotional stability. Furthermore, extraverts were shown to experience greater variability of affective state than other personality traits, and the activation of negative or positive affect reaction may depend on the person’s characteristics of neuroticism as well [35]. Hence, due to the instability of extraverts’ affective reflection, it is difficult to identify whether extraverts would show less decrement performance after negative feedback. Clinically, personality disorders with emotional instability, such as borderline personality, which is characterized by affect and interpersonal dysregulation [36], show disruption in the frontal lobe function [37]. Such disruption affects executive function and reduces the processing speed of the aforementioned tasks in this study. Therefore, emotional support, such as giving advice and reassurance, is more important for extraverts and those showing neuroticism [9]. Research [38,39] has indicated the mediators of the conscientiousness and performance relationship—these include establishing goals, committing to goals, and expectancy of attaining goals. Also, with the tasks being difficult yet attainable, accompanied by feedback, and given that the source of feedback is credible, conscientious people’s motivation and performance are boosted towards the best condition [40]. Therefore, it is assumed that negative feedback is being treated as a challenge by conscientious people, which will incentivize them to improve. However, the current study outcome is not consistent with the previous research. Yet, another study has raised that the effectiveness of the moderating effect of a conscientious personality may depend on the nature of the task, as well as how the feedback was given. Swift and Peterson (2018) have explored a similar relation model as this research did, but included the comparison between different kinds of tasks (playful, neutral, and frustrating puzzle task) [41]. Their research has indicated that conscientious individuals are more sensitive to difficult tasks, and therefore are being more incentivized by negative feedback. However, this circumstance does not apply to playful tasks; conversely, conscientious individuals tend to be overwhelmed by negative feedback on frustrating tasks. 

The acceptance of null hypotheses demonstrated the low representativeness of the sample data in testing the moderating effect of personality traits on feedback and performance. To conclude with the limitations of the current sample, there are a few explanations.

Sample size. One possible reason can be explained by the limitation of the sample size (68). As there was limited research on presenting the exact relationship model, the original effect size was referenced from two related studies separately. This will affect the representativeness of the sample size to a certain extent. Therefore, to improve the representativeness of the sample, a post hoc power calculation was conducted to determine the power given for effect size estimation in the current study. Meanwhile, expecting a fixed regression model in the future, taking the weak R-squared value (R² = 0.043) of the current data, the new sample size was calculated in order to authenticate the most suitable sample size for this research model. This was done using the G*Power calculation method [18], with the standard α = 0.05 and power (1-β) = 0.80 [19] and the new effect size of 0.043. Given the statistical test set as linear multiple regression, the G* Power calculation suggested the new sample size should have 185 people, with the degree of freedom at 182.

The personality inventory. It is impossible to categorize individuals to one particular personality trait. Referring to the personality inventory (IPIP-NEO 120) used in the current research, the results displayed the score of each personality trait, and a person’s personality traits were determined according to the highest score given. However, some individual’s scores may be similar between traits or even scored the same. Based on the multidimensional construct of personality, the characteristics of the personality traits would influence people’s reactions and behaviour differently at different circumstances, causing difficulty for the regression model to reflect the exact effect of personality traits. This phenomenon can be supported by the interactive effect of personality traits. Witt et al. (2002) has supported the interactive effect and demonstrated that highly conscientious individuals did have an interactive effect when they were low in agreeableness, which led them to a lower rating in job performance than those with high agreeableness [27]. Research has also hypothesized that individuals’ stress exposure and coping are dependent on their different personality interactions [42]. For example, we can hypothesize that individuals with high extraversion and high conscientiousness are better in emotion-focused coping, while conversely, high neuroticism–low conscientiousness individuals tend to be dysfunctional in coping with stress [43]. The mixed-effect model has indicated a fixed effect of the personality traits variable, but the data collected did not indicate the personality interaction effect. With the significant results from Grant (2006), the inclusion of all personality combinations could demonstrate a more comprehensive outcome, thus the personality interactive effect hypothesizes should be cultivated. 

The methodological approach. Other than the interactive effect between personality traits, there are other possible factors which will create a certain effect on feedback and performances. Firstly, the study has adopted the convenient sampling method by recruiting participants who the experimenters were familiar with. Yet, different experimenters were involved to reduce response bias. Moreover, the feedback statements adopted were standardized—this had helped to reduce the experimenter effect as well. Secondly, the use of a correlational study would have reduced the representativeness in identifying the significant differences in the performance with and without feedback. Instead, a between-subject study would be an ideal design, in which it can compare the performance between the control (without feedback) and experimental groups (positive feedback group and negative feedback group). In addition to the restriction of a correlational study, a third condition, a repeated Stroop task without feedback, should be done at the end of the experiment. With the neutral condition always first, the performance after feedback was considered as affected by the practice and learning effect. Therefore, by repeating the neutral condition at the end of the experiment, it could infer the effects of feedback and no feedback, as well as reduce the practice effect bias. Thirdly, like the aforementioned, the performance of different personality traits would be influenced by the nature of the task [41]. Thus, it raised the possibility that the feedback on a cognitive task was not influential enough to affect participants’ performance. As a result, a more practical task, such as providing an academic test for students at schools, may be able to reveal the performance effect significantly. This study has another limitation. We only administered the Stanford sleepiness questionnaire. The depression, anxiety, and stress levels of participants were not assessed. Depression is common among the general population in the community [44] and depression is associated with impaired cognitive function [45]. There are other confounders that were not explored in this study. 

Lastly, the recruited sample of this research may be considered as having low external reliability, given that it cannot be applied throughout the country. Taking Scotland’s population structure as an example, until the mid-2017, the percentages of female and male distribution were quite equal, with the highest population proportion at the age range of 45–64 [46]. However, in this research, there was a much higher percentage of females, with the average age range at 25–30. Therefore, it may be considered as being less representative of the general population. 

This research has only demonstrated one of the factors—personality traits that affect the relationship between feedback and performance. Other possible mediating and moderating factors, such as emotions and intrinsic motivators, as mentioned above, as well as retesting with the post-calculated sample size, can be applied in future research. This further implication can enhance the validity and reliability of this relationship model, as well as enhancing the theoretical explanation of it. 

To conclude, the current study demonstrated that personality traits could be a moderating factor on task performance when different types of feedback were given. The results conformed with the concept of behaviourism, in that negative feedback is generally treated as having a negative effect, thus leading to lower task performance. However, this effect was reduced on persons scoring high in extraversion. Still, there were various aspects, such as the personality interaction effect, the sample size, and the confounders to be included, that have affected the generalizability of the research, in which these aspects should be taken into account in future studies.

## Figures and Tables

**Table 1:** Effect of performance feedback.

	Median Mean	t	df	*p*	95% Confidence Interval of the Difference	
					Lower	Upper
Neutral_positive(RT)_ Neutral_negative(RT)	**9**	0.485	69	0.629	−30	49
Positive_RT_ Negative_RT	−9	−0.485	69	0.629	−49	30
Neutral_RT_ Positive_RT	220	9.221	69	0.000	172	268
Neutral_RT_ Negative_RT	221	8.293	69	0.000	160	261

Neutral_Negative(RT) = the median mean differences between no feedback and negative feedback Stroop task performance; Neutral_Positive(RT) = the median mean differences between no feedback and positive feedback Stroop task performance; Neutral_RT = response time without feedback; Positive_RT = response time after receiving positive feedback; Negative_RT = response time after receiving negative feedback (Remarks: a higher mean of median refers to a faster response time after receiving feedback).

**Table 2:** Intercorrelation of variables under the study.

	2	3	4	5	6	7	8	9	10	11	12
1. Neutral_positive	0.673 **	−0.059	−0.037	−0.022	0.156	−0.003	0.205 *	−0.212 *	−0.041	−0.110	0.153
2. Neutral_negative		0.002	−0.019	0.009	0.150	0.046	0.201 *	−0.162	−0.050	−0.162	0.139
3. Age			−0.086	−0.044	−0.303 **	−0.096	0.175	0.292 **	−0.008	−0.051	0.076
4. Gender				0.142	0.001	0.029	−0.097	−0.053	0.044	−0.030	0.011
5. Employment					−0.103	0.393 **	−0.112	−0.268 *	−0.150	−0.089	−0.021
6. Education						0.096	0.089	−0.033	0.136	−0.127	0.294 **
7. Sleepiness							0.003	0.014	−0.098	0.012	0.150
8. Extraversion								0.149	0.315 **	−0.339 **	0.309 **
9. Agreeableness									0.382 **	−0.137	0.167
10. Conscientiousness										−0.235 *	0.163
11. Neuroticism											−0.063
12. Openness											

* Correlation is significant at the 0.05 level (2-tailed). ** Correlation is significant at the 0.01 level (2-tailed. Neutral_Negative = the median differences between no feedback and negative feedback Stroop task performance; Neutral_Positive = the Median differences between no feedback and positive feedback Stroop task performance.

## Appendix A

**Table A1:** Experiment order list.

	Sequence 1	Sequence 2	Sequence 3	Sequence 4
Step 1	Filling in the demographic questions displayed by Open Sesame			
Step 2	Randomized Stroop Task	Randomized Stroop Task	Randomized Stroop Task	Randomized Stroop Task
Step 3	TMT Version A	TMT Version A	TMT Version B	TMT Version B
Step 4	Positive Feedback	Negative Feedback	Positive Feedback	Negative Feedback
Step 5	Randomized Stroop Task	Randomized Stroop Task	Randomized Stroop Task	Randomized Stroop Task
Step 6	TMT Version B	TMT Version B	TMT Version A	TMT Version A
Step 7	Negative Feedback	Positive Feedback	Negative Feedback	Positive Feedback
Step 8	Randomized Stroop Task	Randomized Stroop Task	Randomized Stroop Task	Randomized Stroop Task
Step 9	Complete The International Personality Item Pool Presentation of the NEO PI-R (IPIP-NEO 120) questionnaire			

TMT = Trail Making Test.

**Table A2:** Mean, median, and standard deviation of measuring variables.

	Mean	Median	Std. Deviation
Extraversion	41.88	74	23.64
Agreeableness	51.47	52.5	27.87
Conscientiousness	45.48	46	25.69
Neuroticism	46.41	45	26.07
Openness	39.27	43	24.20
Neutral_RT	1087	952	676.53
Positive_RT	866	748	1458.89
Negative_RT	876	794	2407.02
Neutral_positive(RT)	220	177	200.19
Neutral_negative(RT)	210	153	212.80

Neutral_RT = response time without feedback; Positive_RT = response time after receiving positive feedback; Negative_RT = response time after receiving negative feedback; Neutral_Negative(RT) = the median differences between neutral and negative feedback Stroop task performance; Positive_negative(RT) = the median differences between positive and negative feedback Stroop task performance.

**Table A3:** Regression analysis—the direct effect of personality variables and Stroop performance.

Neutral_RT	Beta	t	*p*
Extraversion	0.194	1.456	0.150
Agreeableness	−0.204	−1.607	0.113
Conscientiousness	−0.093	−0.706	0.483
Neuroticism	−0.142	−1.136	0.260
Openness	0.157	1.268	0.210
**Positive_RT**			
Extraversion	0.147	1.075	0.287
Agreeableness	−0.120	−0.919	0.361
Conscientiousness	−0.096	−0.708	0.481
Neuroticism	−0.138	−1.066	0.291
Openness	0.125	0.981	0.330
**Negative_RT**			
Extraversion	0.159	1.166	0.248
Agreeableness	−0.156	−1.201	0.234
Conscientiousness	−0.076	−0.561	0.577
Neuroticism	−0.105	−0.817	0.417
Openness	0.137	1.083	0.283

Neutral_RT = response time without feedback; Positive_RT = response time after receiving positive feedback; Negative_RT = response time after receiving negative feedback.
